# *Maerua angolensis* DC. (Capparaceae) Stem Bark Extract Protects against Pentylenetetrazole-Induced Oxidative Stress and Seizures in Rats

**DOI:** 10.1155/2018/9684138

**Published:** 2018-05-02

**Authors:** Charles Kwaku Benneh, Robert Peter Biney, Augustine Tandoh, Felix Agyei Ampadu, Donatus Wewura Adongo, Jonathan Jato, Eric Woode

**Affiliations:** ^1^Department of Pharmacology and Toxicology, School of Pharmacy, University of Health and Allied Sciences, Ho, Ghana; ^2^Department of Pharmacology, School of Medical Sciences, University of Cape Coast, Cape Coast, Ghana; ^3^Department of Pharmacology, Kwame Nkrumah University of Science and Technology, Kumasi, Ghana; ^4^Department of Pharmacology, School of Pharmacy, Central University, Accra, Ghana; ^5^Department of Pharmacology, School of Medicine, University of Health and Allied Sciences, Ho, Ghana; ^6^Department of Pharmacognosy, School of Pharmacy, University of Health and Allied Sciences, Ho, Ghana

## Abstract

**Introduction:**

The stem bark of* Maerua angolensis* DC. (Capparaceae) is traditionally used for management of epilepsy. Our aim was to evaluate the antiseizure potential and identify possible mechanisms by which the effects are registered.

**Methods:**

The petroleum ether/ethyl acetate extract (100–1000 mg kg^−1^) was administered per os to male Sprague-Dawley rats after pretreatment with flumazenil (0.3 mg kg^−1^) or L-arginine (150 mg kg^−1^) or sildenafil (5 mg kg^−1^) and they subsequently received a subcutaneous injection of pentylenetetrazole (65 mg kg^−1^). Rats were observed for latency to and duration of myoclonic seizures and additionally the level of protection against oxidant markers and products was assessed* in vitro* and* in vivo*.

**Results:**

The extract (300 and 1000 mg kg^−1^, p.o.) significantly delayed the onset and decreased the duration and frequency of PTZ-induced convulsions. The anticonvulsant effect of MAE (300 mg kg^−1^, p.o.) was reversed by pretreatment with flumazenil, L-arginine, or sildenafil. Also, MAE (300 mg kg^−1^) treatment reversed significantly PTZ-induced oxidative stress in rat brain tissue.

**Conclusion:**

The petroleum ether/ethyl acetate fraction exhibits antiseizure activity by affecting GABAergic and nitric oxide-cGMP pathways. In addition, the extract protects against the generation of free radicals and the oxidative products of the PTZ-induced seizures.

## 1. Introduction

An epileptic seizure is a transient occurrence of signs and/or symptoms due to abnormal excessive or synchronous neuronal activity in the brain [1]. In epilepsy, there is a characteristic predisposition to generate seizures with accompanying neurobiological and cognitive disturbances. An appreciable number of current antiepileptic agents modulate GABA_A_ receptor or enhance the response of this receptor to the gamma amino butyric acid (GABA). Additionally, dysfunctional nitric oxide (NO) pathways have also been implicated in the pathophysiology and progression of experimentally induced epilepsy [2].

An additional mechanism that has received significant attention by researchers is oxidative stress and its contribution to the generation and prognosis of epilepsy [3, 4]. Although the influence of oxidative stress as a cause or consequence of epilepsy is still debated, it is agreed as common knowledge that the production of free radicals plays a role in the pathogenesis of epilepsy [5]. Experimental studies suggest that an imbalance in the oxidative state of brain neuronal cells is a contributing factor to the induction and propagation of epilepsy. The brain is particularly vulnerable to oxidative stress. Comparatively high production of reactive oxygen species and ironically low antioxidant capacity make it exceptionally vulnerable to neuronal and glial cell damage, which in turn can contribute to the initiation and progression of several central nervous disorders including epilepsy [3].

Prooxidative conditions including seizures lower antioxidant defenses and can predispose the brain to oxidative stress. It has therefore been postulated that agents that possess significant* in vivo* antiepileptic activity by acting on the known receptors mediating the progression of epilepsy (e.g., GABA_A_ receptor) and simultaneously have significantly high* in vivo* antioxidant activity might positively affect the prognosis of the condition comparatively to a greater extent than an agent that only has comparable efficacy as a receptor modulator [3]. Thus, the need to source for clinically efficacious and safer antiepileptic drugs (AEDs) with such improved mechanisms and clinical benefit is still valid.

The plant* Maerua angolensis* DC. (Capparaceae) has been used for a myriad of therapeutic benefits in the West African subregion [6–8]. Its antiepileptic and antischizophrenic effects are chief among the traditional use claims in Ghana. The roots and stem bark decoction of the stem bark of* Maerua angolensis *DC. have been used in Tanzania for the management of epilepsy [9]. Previous medicinal plant surveys by Chhabra et al. [6] and Burkill [10] indicate that the stem bark and the leaves have been traditionally used to manage epilepsy. Irrespective of these claims, there is currently little scientific evidence to support this traditional use.

The aim of this study was therefore to investigate whether* Maerua angolensis* DC. has a preventive effect against PTZ-induced seizure and postseizure-induced oxidative stress. Recently, we have demonstrated that the anxiolytic effect of* Maerua angolensis *DC. stem bark extract, in zebrafish, is mediated by GABAergic and 5-HT systems [11]. We assessed the possible involvement of GABA_A_ receptor modulation in its antiseizure effects. Additionally, the involvement of NO-cGMP system(s) in the PTZ-induced seizure model using sildenafil and L-arginine was explored. To identify the potential antioxidant benefit, the extract might have on the prognosis in the PTZ-induced seizure in the rat model an assessment of* in vitro* protection against free radicals and an* in vivo* assessment of the oxidative state and markers after PTZ-induced seizures were assessed.

## 2. Methods

### 2.1. Plant Extraction and FT-IR Analysis of Crude Extract

The stem bark of* Maerua angolensis *DC. was collected from Kwahu Tafo in the Eastern region of Ghana (6.415360°N, 0.363160°W) and authenticated at the Department of Herbal Medicine, Faculty of Pharmacy and Pharmaceutical Sciences, Kwame Nkrumah University of Science and Technology. A voucher specimen (KNUST/FP/12/051) has been kept at the herbarium of the faculty.

The dried stem bark was chopped and pulverized into a fine light-brown powder. Approximately 1.8 kg of the powder was then extracted by cold percolation with petroleum ether and ethyl acetate mixture (50 : 50). The solvent mixture was drained 72 h later to obtain a dark green extract that was further concentrated in a rotary evaporator at 60°C and under reduced pressure. The concentrate obtained was further dried in a hot air oven at 55°C for 72 h to obtain a green semisolid mass (~8.5 g), which was then stored in a freezer at −40°C until use. The spectral region between 400 and 1400 cm^−1^ is usually considered as the unique region for every compound/compound mixtures and hence can be used for identification and quality control. Hence, triplicate FT-IR (PerkinElmer® UATR Two) spectra were subsequently generated for the extract.

#### 2.1.1. Chemicals and Drugs

Sildenafil was obtained from Pfizer Inc., Brooklyn, NY, USA, diazepam from Intas, Gujarat, India, flumazenil from Roche Pharmaceutical Ltd., Garden City, UK, adrenaline from Indus Pharma Pvt., India, and EDTA-free protease inhibitor cocktail from Santa Cruz Biotechnology, Inc., Dallas, Texas, USA. Pentylenetetrazole, trichloroacetic acid, thiobarbituric acid, sodium dodecyl sulphate, sodium chloride, Tween 80, n-butanol, Triton-X, L-arginine, DTNB (5,5′-Dithiobis (2-nitrobenzoic acid)), nitro blue tetrazolium (NBT), phenazine methosulfate (PMS), ethylenediaminetetraacetic acid (EDTA), DPPH (2,2-diphenyl-1-picrylhydrazyl), NADH (nicotinamide adenine dinucleotide), and Tris-HCl were obtained from Sigma-Aldrich, Inc., St. Louis, MO, USA.* Maerua angolensis* extract (MAE) was prepared by solubilizing with Tween 80 q.s.

### 2.2. Animals

Sprague-Dawley rats (150–200 g) were obtained from the animal house of the Department of Pharmacology, Kwame Nkrumah University of Science and Technology, Kumasi, Ghana. Rats were housed in groups of 3 in stainless steel cages (34 × 47 × 18 cm^3^) with soft wood shavings as bedding and housing conditions as follows: temperature was maintained at 23–25°C and relative humidity was 60–70% in a 12 h light-dark cycle. They had free access to tap water and food (commercial pellet diet, GAFCO, Tema, Ghana). The studies were conducted in accordance with accepted principles for laboratory animal use and care (NRC, 2010). Approval for this study was obtained from the faculty's Ethics Committee.

### 2.3. PTZ-Induced Convulsions in Rats

Pentylenetetrazole (65 mg kg^−1^, s.c.) was used to induce clonic convulsions. Rats were divided into seven groups and received MAE (100, 300, 1000 mg kg^−1^, p.o.), diazepam (0.1, 0.3, and 1 mg kg^−1^, i.p.), or normal saline (10 mL kg^−1^ i.p.) 30 min (i.p.) or 1 h (p.o.) before the injection of PTZ, respectively. After PTZ injection, animals were placed in testing chambers (15 × 15 × 15 cm^3^). A mirror angled at 45° below the floor of the chamber allowed a complete view of the convulsive event. Behaviour of the animals was captured with a camcorder for a 30-minute period after PTZ challenge and the latency to myoclonic jerks and the incidence of generalized tonic-clonic convulsions were recorded using a camcorder and analyzed using the behavioural analysis software, JWatcher® version 1.0 (University of California, Los Angeles, USA, and Macquarie University, Sydney, Australia; available at http://www.jwatcher.ucla.edu/).

### 2.4. Involvement of GABAergic System

To investigate the involvement of GABA_A_ receptor in the anticonvulsant effects of MAE, rats (*n* = 7) were pretreated with flumazenil (2 mg kg^−1^ i.p.) followed by a single diazepam (0.3 mg kg^−1^ i.p.) dose or three-day MAE (300 mg kg^−1^ p.o.) treatment and then challenged, 30 min after diazepam and 60 min after the last MAE treatment, with a single subcutaneous dose of PTZ (65 mg kg^−1^) to induce clonic convulsions. The latency, frequency, and duration of clonic convulsions were assessed for a 30 min period using JWatcher version 1.0.

### 2.5. Involvement of Nitric Oxide Pathway

Rats that received MAE (300 mg kg^−1^) were orally dosed three consecutive days before the mechanistic study was performed. To investigate the involvement of L-arginine-NO-cGMP pathway, rats (*n* = 7) were pretreated with L-arginine (150 mg kg^−1^ i.p.) or sildenafil (5 mg kg^−1^ i.p.) or vehicle 15 minutes before the last MAE (300 mg kg^−1^ p.o.) treatment. Treated animals were challenged with PTZ (65 mg kg^−1^) 45 min after the last MAE treatment administration. The latency, frequency, and duration of clonic convulsions were assessed for a period of 30 min using JWatcher version 1.0.

### 2.6. *In Vitro* Antioxidant Assay

#### 2.6.1. DPPH Radical Scavenging Activity

The method of Blois [12] with modification was used for the determination of scavenging activity of DPPH free radical. DPPH (1.0 mL, 0.135 mM) prepared in absolute methanol was mixed with 1.0 mL of dissolved extracts and standards (ascorbic acid) of concentration ranging from 20 to 2000 *μ*g mL^−1^. The reaction mixture was shaken vigorously and left in the dark at room temperature for 30 minutes. The absorbance was measured spectrophotometrically at 517 nm against a reagent blank containing only methanol. All experiments were conducted in triplicate. Control experiment contained only methanol and DPPH free radical. The percentage scavenging activity of DPPH radical was calculated using the following formula:

(1) %  scavenging  activity  of  DPPH=Acontrol−AsampleAcontrol×100%,

where *A*_control_ is the absorbance of the control reaction and *A*_sample_ is the absorbance in the presence of the sample extracts or standard.

#### 2.6.2. Superoxide Anion Scavenging Activity

The superoxide anion scavenging activity was measured as described by Robak and Gryglewski [13]. The superoxide anion radicals were generated in 3.0 mL of Tris-HCl buffer (16 mM, pH 8.0), containing 0.5 mL of NBT (0.3 mM), 0.5 mL NADH (0.936 mM) solution, 1.0 mL MAE or ascorbic acid (20–2000 *μ*g mL^−1^) in Tris-HCl, and 0.5 mL Tris-HCl buffer (16 mM, pH 8.0). The reaction was started by adding 0.5 mL PMS solution (0.12 mM) to the mixture and incubated at 25°C for 5 minutes and then the absorbance change was measured at 560 nm against a blank sample containing Tris-HCl only. Ascorbic acid was used as the standard antioxidant. The decrease in absorbance at 560 nm as concentration increases was measured against an appropriate blank to determine the quantity of formazan generated. The percentage of inhibition of superoxide anion was calculated using the following equation:

(2) $$\begin{array}{c} \%\;\;inhibition=\frac{A_{control}-A_{sample}}{A_{control}}\times 100\%, \end{array}$$

where *A*_control_ is the absorbance of the control reaction and *A*_sample_ is the absorbance in the presence of the sample extracts or standard.

#### 2.6.3. Estimation of Antilipid Peroxidation

A modified thiobarbituric acid-reactive species (TBARS) assay described by Dasgupta and De [14] was used to measure the lipid peroxide formed using egg-yolk homogenates as lipid-rich media.

About 0.5 mL of egg yolk homogenate (10% v/v in distilled water) and 0.1 mL of the extract and standard (20–2000 *μ*g mL^−1^) were mixed separately in a test tube and the volume was made up to 1 mL with distilled water. Finally, 0.05 mL Fe_2_SO_4_ (0.07 M) was added to the mixture and incubated for 30 minutes to induce lipid peroxidation. Thereafter, 1.5 mL of 20% acetic acid (pH adjusted to 3.5 with NaOH), 1.5 mL of 0.8% w/v thiobarbituric acid (TBA) (prepared in 1.1% sodium dodecyl sulphate), and 0.05 mL 20% w/v trichloroacetic acid (TCA) were added, vortexed, and heated in a water bath at 100°C for 1 h. To eliminate this non-MDA interference, another set of samples was treated in the same way, but incubating without TBA, so as to subtract the absorbance of the non-MDA interference from the test and standards absorbance. After cooling, the coloured TBA-MDA complex was extracted with 5 ml n-butanol by vigorous shaking and centrifuging at 3000 ×g for 10 min.

The absorbance of the organic upper layer was measured at 532 nm. For control, 0.1 mL of distilled water was used in place of the extract or standard:

(3) %  antilipid  peroxidation=Acontrol−AsampleAcontrol×100%,

where *A*_control_ is the absorbance of the control reaction and *A*_sample_ is the absorbance in the presence of the sample extracts or standard.

### 2.7. *In Vivo* Antioxidant Assay

#### 2.7.1. Tissue Preparation

Treated rats from the PTZ-induced convulsion experiment above (Section 2.3) were euthanized and the brains were quickly excised, washed with normal saline, and stored at −80°C. On the day of the experiment, the brain tissue was thawed, weighed, and homogenized in phosphate buffer to obtain 10% w/v of homogenate. The tissue homogenate obtained was used to assay superoxide dismutase activity. The supernatant obtained after centrifuging at 20,000 ×g for one hour was assayed for catalase levels and the degree of lipid peroxidation.

Each assay below was carried out in a single unused 96-round-well plate (Nunc^TM^, Thermo Fischer Scientific, Waltham, MA, USA). Absorbance was measured with Synergy H1® hybrid plate reader at wavelengths specified below. Triplicate readings were taken for each treatment sample and the direct absorbances or derived data was represented as mean ± SEM.

The protein content of the brain homogenates was assayed using Flexor EL150TM (Vital Scientific) biochemistry analyzer. All readings were normalized with the protein content of the respective tissue sample.

#### 2.7.2. Lipid Peroxidation

The LPO level was estimated in brain tissue supernatant in terms of malondialdehyde (MDA) which was determined as per the methods of Heath and Packer [15]. MDA content was estimated by addition of 3 ml trichloroacetic acid (20% w/v) containing thiobarbituric acid (0.5% w/v) to 1 ml of tissue supernatant.

The mixture was heated at 95°C for 30 min and then quickly cooled in an ice bath. The tube was centrifuged at 10,000 ×g for 10 min, and then absorbance of the supernatant was read at 532 nm. The value for the nonspecific absorption at 600 nm was subtracted from the 532 nm reading. The concentration of MDA was calculated using MDA's extinction coefficient of 155 mM^−1 ^cm^−1^.

#### 2.7.3. Superoxide Dismutase

Brain superoxide dismutase activity was measured based on the ability of SOD to inhibit autoxidation of adrenaline to adrenochrome. To 0.5 ml of tissue homogenate, 0.75 ml of ethanol (96% v/v) and 0.15 ml of chilled chloroform were added. The resulting solution was centrifuged at 2000 ×g for 20 minutes (30°C) to obtain a clear supernatant. An aliquot of 0.5 ml of EDTA (0.6 mM) was added to the supernatant solution containing 1 mL of bicarbonate buffer (0.1 M, pH 10.2). A 50 *μ*L aliquot of the adrenaline solution (1.3 mM) was added to the mixture to initiate the reaction and the absorbance of the adrenochrome formed was measured at 480 nm at 25°C. The absorbance of a sample blank containing all reagents apart from tissue homogenate was also measured at 480 nm.

#### 2.7.4. Catalase Activity

The catalase activity was assayed colorimetrically at 620 nm as described by the method of Aebi [16]. It is based on the ability of the enzyme source to break down H_2_O_2_. An aliquot of 100 *μ*L of the supernatant was added to 130 *μ*L of potassium buffer (50 mM, pH = 7.0). The reaction was initiated in well by the addition of 65 *μ*L of H_2_O_2_ (10 mM) and the absorbance of the resulting mixture was read at 620 nm with an appropriate blank.

### 2.8. Statistics

Data are presented as mean ± SEM. Data were analyzed using one-way analysis of variance (ANOVA). When ANOVA was significant, multiple comparisons between treatments were done using Sidak post hoc test. Significance between grouped data was assessed using a two-way ANOVA followed by a Sidak post hoc test. *P* values less than or equal to 0.05 were considered significant. Dose-responses curves are constructed using iterative curve fitting with the following nonlinear regression (three-parameter logistic) equation:

(4) $$\begin{array}{c} Y=\frac{a+\left(b-a\right)}{\left(1+10^{\left(\log ED_{50}-X\right)}\right)}, \end{array}$$

where *X* is the logarithm of dose and *Y* is the response. *Y* starts at *a* (the bottom) and goes to *b* (the top) with a sigmoid shape. The fitted midpoints (ED_50_) of the curves were compared statistically using *F* test with GraphPad Prism for Windows version 6.01 (GraphPad® Software, San Diego, CA, USA).

## 3. Results

### 3.1. FT-IR Analysis of Crude Extract

**Figure 1:**
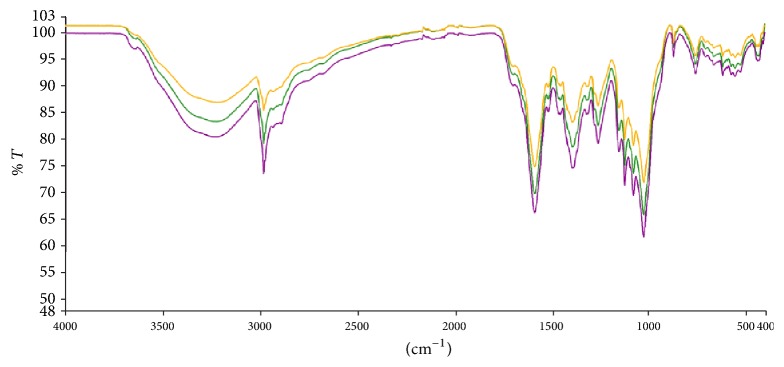
Baseline corrected Infrared spectra of the petroleum ether/ethyl acetate fraction of* Maerua angolensis* stem bark extract. The experiment was repeated thrice, with similar conditions, from 400 to 4000 cm^−1^. Peak values and labels are available in Table 1 and Figure 9.

Infrared spectra analysis is a quick and useful tool used in the fingerprinting of complex mixtures such as plant extract. The characteristic peaks and corresponding absorbances can be used as a unique reference for future comparison of complex mixtures that have not yet been characterized. This study generated fingerprint spectra (Figure 1) in the region from 400 to 1400 cm^−1^ for subsequent comparison.

### 3.2. PTZ-Induced Convulsions in Rats

#### 3.2.1. Pentylenetetrazole-Induced Convulsion in Rats

In the pentylenetetrazole-induced convulsions, reductions in the frequency and duration of convulsions signify anticonvulsant properties of the test compound or extract. Additionally, a delay in the onset of the convulsions can also be used as an indicator of anticonvulsant effect.

**Figure 2:**
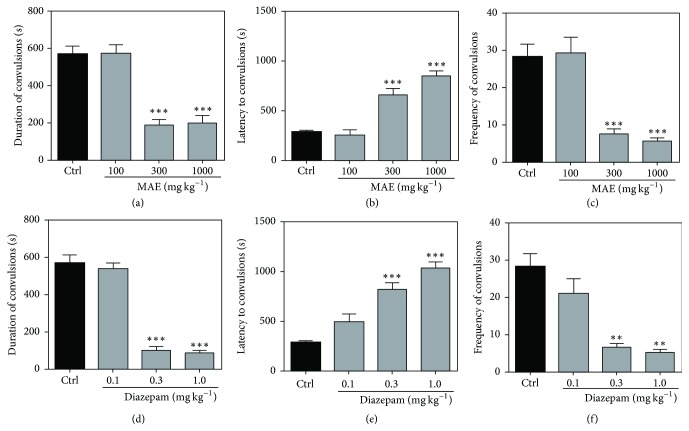
Effects of MAE (100−1000 mg kg^−1^) and diazepam (0.1–1.0 mg kg^−1^) on the duration ((a) and (d)) and latency to convulsion ((b) and (e)) and number of clonic convulsions ((c) and (f)) for a 30-minute test period in the pentylenetetrazole-induced convulsions. Data are expressed as group mean ± SEM. ^*∗∗*^*P* < 0.01 and ^*∗∗∗*^*P* < 0.001 indicate significant difference compared to control (one-way ANOVA followed by Sidak post hoc test).

In this test, the extract significantly delayed the onset of clonic convulsions with statistical significance at 300 and 1000 mg kg^−1^ (*P* < 0.001; Figure 2(b)). Additionally, a one-way ANOVA revealed that MAE significantly reduced the frequency (*P* < 0.001 at 300 and 1000 mg kg^−1^; Figure 2(c)) and duration (*P* < 0.001 at 300 and 1000 mg kg^−1^; Figure 2(a)) of clonic convulsions.

Diazepam, the reference anticonvulsant, delayed the onset of clonic convulsions (0.3 and 1.0 mg kg^−1^; *P* < 0.001; Figure 2(e)). Also, diazepam caused a significant reduction in the frequency (*P* < 0.01; Figure 2(f)) and duration (*P* < 0.0001; Figure 2(d)) of clonic convulsions.

#### 3.2.2. Involvement of GABAergic System

**Figure 3:**
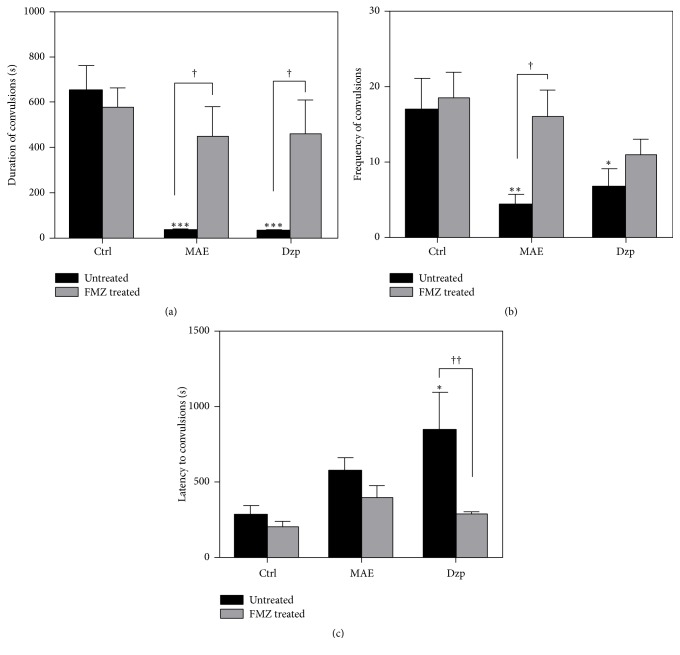
Effects of MAE (300 mg kg^−1^) and diazepam (0.3 mg kg^−1^) on the (a) duration, (b) latency, and (c) number of clonic convulsions for a 30-minute period in the PTZ-induced convulsions after flumazenil treatment. Data are expressed as group mean ± SEM. ^*∗*^*P* < 0.05, ^*∗∗*^*P* < 0.01, and ^*∗∗∗*^*P* < 0.001 indicate significant difference compared to control (one-way ANOVA followed by Sidak post hoc test). ^†^*P* < 0.05 and ^††^*P* < 0.01 indicated significant difference when treated and untreated rats were compared (two-way repeated-measures ANOVA followed by Bonferroni's post hoc test).

Figures 3(a)–3(c) show that pretreatment with flumazenil (2 mg kg^−1^, i.p.) had no effects on latency, duration, and frequency of convulsions as compared with vehicle-treated animals. Subacute MAE (300 mg kg^−1^, p.o.) treatment significantly decreased both frequency (*P* < 0.01) and duration (*P* < 0.001) of clonic convulsions (Figures 3(a) and 3(b)). However, pretreatment with flumazenil significantly reversed the effect of MAE (300 mg kg^−1^, p.o.) by increasing duration (*P* < 0.05) and frequency (*P* < 0.05) of clonic seizures induced by PTZ. Similar results were obtained for diazepam.

#### 3.2.3. Involvement of NO-cGMP Pathway

**Figure 4:**
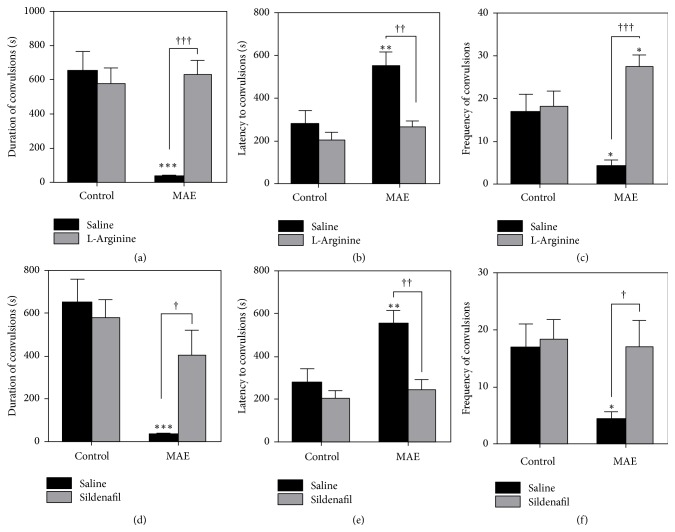
Effects of MAE (300 mg kg^−1^) on the ((a) and (d)) duration, ((b) and (e)) latency, and ((c) and (f)) number of clonic convulsions for a 30-minute test period in the pentylenetetrazole-induced convulsions after pretreatment with L-arginine ((a), (b), and (c)) and sildenafil ((d), (e), and (f)). Data are expressed as group mean ± SEM. ^*∗*^*P* < 0.05, ^*∗∗*^*P* < 0.01, and ^*∗∗∗*^*P* < 0.001 indicate significant difference compared to control (one-way ANOVA followed by Sidak post hoc test). ^†^*P* < 0.05, ^††^*P* < 0.01, and ^†††^*P* < 0.001 indicate significant difference when treated and untreated rats were compared (two-way repeated-measures ANOVA followed by Bonferroni's post hoc test).

Nitric oxide (NO) plays a role in the anticonvulsant effects of some antiseizure drugs. The downstream effect of NO results in the elevation of cGMP, which in turn mediates the effects. A precursor of NO synthesis and an inhibitor of cGMP breakdown, l-arginine and sildenafil respectively, can therefore elevate the NO levels and/or activity. Coadministration of pentylenetetrazole after pretreatment with subeffective doses of l-arginine or sildenafil reversed the anticonvulsant effects of MAE. An agent that augments NO production or the downstream effects of subeffective doses of l-arginine or sildenafil can reverse the anticonvulsant effect due to the possible induction of NO-induced excitotoxicity. In this test, subacute treatment with MAE (300 mg kg^−1^ p.o.) alone significantly increased latency and decreased both frequency and duration of the PTZ-induced clonic convulsions (Figures 4(a)–4(c)). Administration of l-arginine (150 mg kg^−1^, i.p., a precursor of nitric oxide) alone had no anticonvulsant effects compared with saline-treated control (Figure 4(a)). A reversal of the anticonvulsant effects of MAE (300 mg kg^−1^, p.o.) was observed after pretreatment with l-arginine or sildenafil. Pretreatment with l-arginine or sildenafil significantly inhibited the anticonvulsant effect of MAE (300 mg kg^−1^, p.o.) by decreasing latency and increasing frequency and duration of clonic seizures as revealed by post hoc analysis.

### 3.3. *In Vitro *Antioxidant Assay

#### 3.3.1. DPPH Radical Scavenging Activity

**Figure 5:**
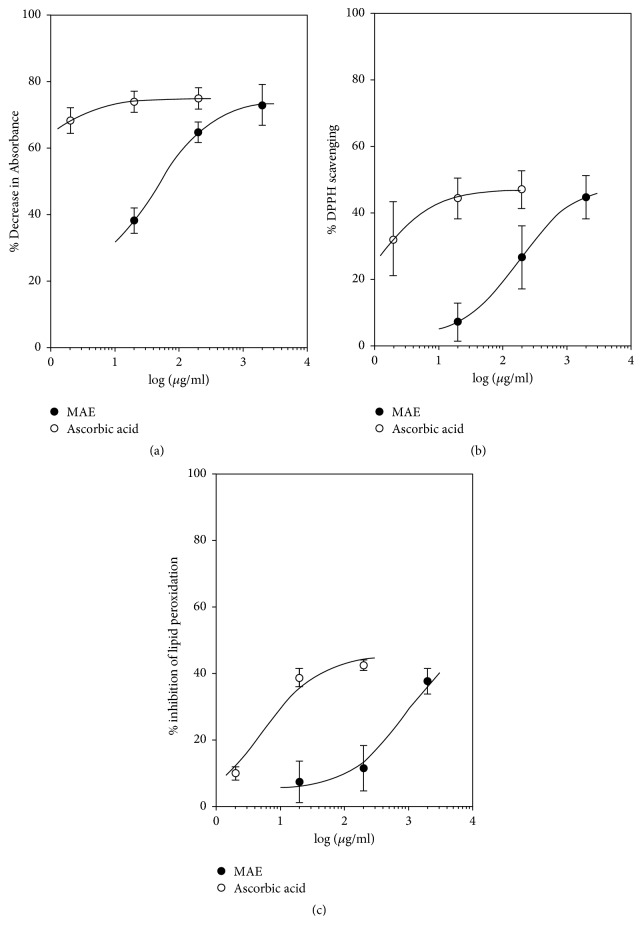
Dose-response curves for MAE (20–2000 *μ*g mL^−1^) and ascorbic acid (2–200 *μ*g mL^−1^) with respect to (a) superoxide scavenging activity, (b) DPPH radical scavenging activity, and (c) antilipid peroxidation activity. Each point represents the mean ± SEM (*n* = 3).

MAE (20–2000 *μ*g mL^−1^) and ascorbic acid (2–200 *μ*g mL^−1^) exerted a concentration-dependent radical scavenging activity (Figure 5(b)). *E*_max_ of MAE (48.46 ± 3.65%) and ascorbic acid (46.95 ± 2.20%) indicates similar efficacies at tested doses. However, the IC_50_ values (*μ*g mL^−1^) suggest that the potency of MAE (181.70 *μ*g mL^−1^) is lower than ascorbic acid (0.93 *μ*g mL^−1^).

#### 3.3.2. Superoxide Anion Scavenging Activity

Similar to DPPH radical scavenging activity, MAE (20–2000 *μ*g mL^−1^) exerted comparable superoxide scavenging activity to ascorbic acid (2–200 *μ*g mL^−1^). *E*_max_ of MAE (74.13 ± 8.19%) and ascorbic acid (74.92 ± 1.03%) indicates similar efficacies at tested doses (Figure 5(a)). The IC_50_ values (*μ*g mL^−1^), however, show that the potency of MAE (4.36 *μ*g mL^−1^) is lower than ascorbic acid (0.72 *μ*g mL^−1^).

#### 3.3.3. Estimation of Antilipid Peroxidation

Tested concentrations of MAE and ascorbic acid showed an appreciable reduction in the formation of thiobarbituric acid-reactive species from the yolk substrate (Figure 5(c)). *E*_max_ of MAE (50 ± 0.00%) and ascorbic acid (45.44 ± 1.17%) indicates similar efficacies at tested doses. The IC_50_ values (*μ*g mL^−1^), similar to the other tests, suggest that the potency of MAE (824.50 *μ*g mL^−1^) is lower than ascorbic acid (0.54 *μ*g mL^−1^).

### 3.4. *In Vivo *Antioxidant Assay

#### 3.4.1. Lipid Peroxidation

**Figure 6:**
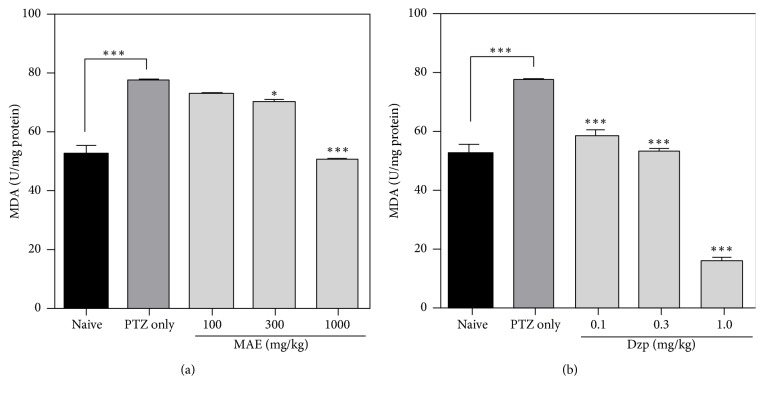
Effects of (a) MAE (100–1000 mg kg^−1^) and (b) diazepam (0.1–1.0 mg kg^−1^) treatment on whole brain lipid peroxidation after PTZ-induced seizures. Data are expressed as group mean ± SEM. ^*∗*^*P* < 0.05 and ^*∗∗∗*^*P* < 0.001 indicate significant difference compared to PTZ group (one-way ANOVA followed by Sidak post hoc test).

Pentylenetetrazole (65 mg kg^−1^ s.c.) increased (*P* < 0.001) the formation of MDA content, which is consistent with other research works, where an increase in reactive oxygen species enhances the production of lipid peroxidation products. Rats treated with MAE (*F*_4,15_ = 49.40; *P* < 0.0001) or diazepam (*F*_4,15_ = 112.2; *P* < 0.0001) reduced thiobarbituric acid reactive substance levels as compared to the PTZ only group (Figures 6(a) and 6(b)).

#### 3.4.2. Superoxide Dismutase

**Figure 7:**
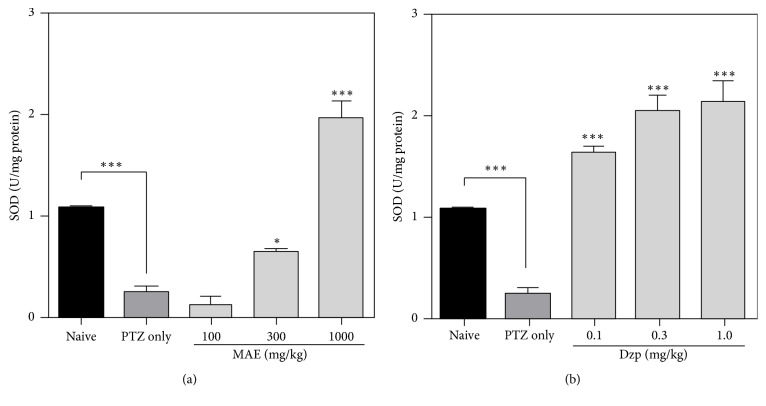
Effects of (a) MAE (100–1000 mg kg^−1^) and (b) diazepam (0.1–1.0 mg kg^−1^) treatment on whole brain superoxide dismutase levels after PTZ-induced seizures. Data are expressed as group mean ± SEM. ^*∗*^*P* < 0.05 and ^*∗∗∗*^*P* < 0.001 indicate significant difference compared to PTZ group (one-way ANOVA followed by Sidak post hoc test).

Subcutaneous injection of pentylenetetrazole (65 mg kg^−1^ s.c.) decreased brain superoxide dismutase activity (*P* < 0.001). The opposite is true for rats treated with 300 and 1000 mg kg^−1^ MAE (*F*_4,13_ = 68.99; *P* < 0.0001) or all tested doses of diazepam (*F*_4,13_ = 34.35; *P* < 0.0001) (Figures 7(a) and 7(b)).

#### 3.4.3. Catalase Activity

**Figure 8:**
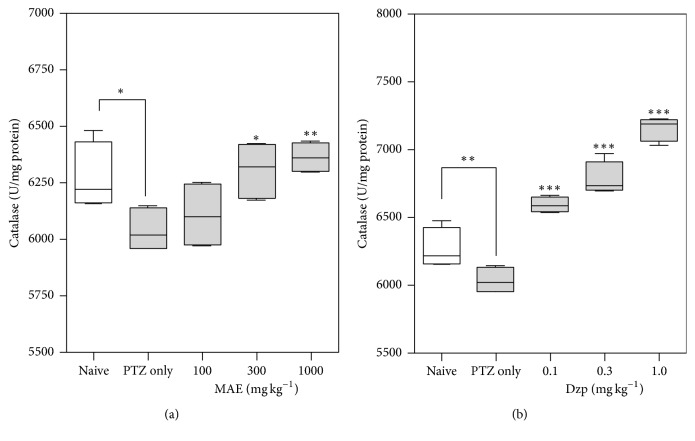
Effects of (a) MAE (100–1000 mg kg^−1^) and (b) diazepam (0.1–1.0 mg kg^−1^) treatment on whole brain catalase levels after PTZ-induced seizures. The lower and upper margins of the boxes represent 25th and 75th percentiles with the extended arms representing the 10th and 90th percentiles, respectively. ^*∗*^*P* < 0.05, ^*∗∗*^*P* < 0.01, and ^*∗∗∗*^*P* < 0.001 indicate significant difference from control compared to PTZ group (one-way ANOVA followed by Sidak post hoc test).

Similar to superoxide dismutase activity in the brain tissue, the activity level of catalase was significantly reduced (*P* < 0.05), compared to PTZ-naïve rats, after subcutaneous injection of pentylenetetrazole (65 mg kg^−1^ s.c.) only. However, rats pretreated with MAE or diazepam showed no reduction in catalase activity (Figures 8(a) and 8(b)). On the contrary, MAE (*F*_4,15_ = 5.110; *P* = 0.0084) and diazepam (*F*_4,15_ = 65.43; *P* < 0.0001) pretreatment dose-dependently increased catalase activity as compared to the PTZ only group.

## 4. Discussion

In recent times, quite a number of Ghanaian plants have been shown to exhibit diverse central nervous system activities ranging from anxiolytic activity to antidepressant and anticonvulsant activity [11, 17–19]. Prior to the working with the petroleum ether/ethyl acetate fraction of the stem bark* (Maerua angolensis)*, FT-IR spectra were generated. These spectra are intended to be a tool of comparison for subsequent work with the extract MAE.

The present study demonstrates that the lipophilic fraction of the stem bark extract significantly reduces pentylenetetrazole-induced seizure outcomes due to interaction with the GABA_A_ receptor and nitricoxidergic system. The GABAergic mediation of antiseizure activity is in agreement with earlier studies carried out with the ethanolic fraction of the stem bark [20].

The GABAergic system is implicated in epilepsy, since enhancement and inhibition of the neurotransmission of GABA will attenuate and enhance convulsion, respectively [21, 22]. PTZ blocks GABA-mediated Cl^–^ influx through an allosteric interaction in the Cl^–^ channel, thus leading to induction of convulsions in animals [23, 24]. Since the effect of PTZ is through GABA-mediated antagonism, it was posited that the protection may be due to an agonistic interaction with the GABA_A_ receptor/system either directly or indirectly. In this study, acute administration of MAE and diazepam exhibited anticonvulsant activity against PTZ-induced seizures by significantly delaying the occurrence of clonic seizures. In addition, MAE decreased the frequency and duration of the clonic seizures. The effect of MAE in PTZ-induced seizure models suggests that its anticonvulsant action is due to its interference with GABAergic system. To further confirm the possible contribution of GABAergic system in the anticonvulsant activity of MAE, flumazenil, a specific antagonist of the GABA_A_ receptor complex [25], was used in antiseizure mechanistic studies. Pretreatment with flumazenil reversed the anticonvulsant effect of MAE, suggesting involvement of GABA_A_ receptor in its anticonvulsant effects. This further confirms the GABA enhancing activity of the MAE.

Evidence gathered over the past decade suggests the involvement of nitric oxide (NO) in the onset, intensity, and progression of seizures, including PTZ-induced seizures [19, 26, 27]. Although some conflicting data exist, most works suggest that decreased nitric oxide production leads to anticonvulsant effects, while increased NO levels produce proconvulsant effects in PTZ-induced seizures [2, 19, 27]. These effects were further corroborated by works done by Akula and colleagues [28], where the authors observed potentiating effects of anticonvulsant activity of subeffective doses of adenosine after pretreatment with l-NAME (a nonselective nitric oxide synthase inhibitor) and a reversal of effect after pretreatment with L-arginine (a precursor of nitric oxide) or sildenafil (a phosphodiesterase V enzyme inhibitor). Nitric oxide in higher concentrations can act as an excitatory proconvulsant in the CNS [27]. In PTZ-induced seizures, NO levels are elevated and hence NOs inhibitors have been found to suppress seizure parameters including latency to convulsions [2, 26, 29]. By extension, elevated levels of NO produced by substrates of the neuronal nitric oxide synthase (nNOs) system can increase the liability to seizure in the CNS after injection of a proconvulsant agent. Additionally, agents that enhance the downstream activity of NO, by preventing the breakdown of cGMP, can enhance the proconvulsant effects of suboptimal levels of NO [28]. In this study, subeffective doses of L-arginine (a precursor of NO) and sildenafil (enhancer of cGMP) activity did not affect seizure parameters measured: latency, frequency, and duration of convulsions. However, the sun-effective dose of L-arginine reversed the anticonvulsant activity of an anticonvulsant dose of MAE. This reversal suggests that MAE alone can produce or enhance the production of NO, by neuronal NOs, which in turn adds on to the NO levels produced by l-arginine-NOs system, leading to a state of excitotoxicity and complete reversal of MAE anticonvulsant activity. It is therefore suggested that MAE produces its ant-convulsant activity in Sprague-Dawley rats by interacting with the L-arginine-NO-cGMP pathway possibly secondary to GABA_A_ receptor activation. Sildenafil is an inhibitor of PDE5, an enzyme that actively breaks down cAMP to yield cGMP [30]. Since a downstream effect of nitric oxide is the production of cGMP via Guanylate cyclase [31], an increase in the cGMP levels further potentiates nitric oxide-mediated effects. The anticonvulsant effect of MAE, in this study, was significantly reversed by a pretreatment with sildenafil, further suggesting that MAE interacts with the nitricoxidergic pathway by increasing cGMP levels either directly or indirectly.

The brain expends about 20% of the oxygen utilized by the body with a corresponding increase in reactive oxygen species (ROS). The lipid-rich nature and the relatively lower level of SOD, catalase, and GSH make the brain exceptionally vulnerable to oxidative stress, making detoxification of ROS an essential task to protect neuronal and glial cells [31]. Oxidative stress is gaining ground as a possible contributor to dysregulation underlying various pathologies such as anxiety and epilepsy. Under oxidative stress, the lipid-rich constitution of the brain can undergo lipid peroxidation, which can result in decreased membrane fluidity and altered neurotransmission.

In oxidative stress, free radicals such as superoxide anions are generated and overwhelm the normal protective mechanisms and consequently contribute to the detrimental effects on brain tissue. Similar to the detrimental effects of superoxide anions, lipid peroxidation damages tissues, specifically membrane lipids. This study indicates that the petroleum ether/ethyl acetate fraction of* Maerua angolensis *DC. possesses significant* in vitro* activity in the DPPH radical scavenging activity, superoxide anion scavenging activity, and thiobarbituric acid-reactive species assay. The antioxidant property of MAE was further confirmed* in vivo *by assessing the degree of protection of the brain after administration of PTZ in rats. It has been established that PTZ-induced seizure increases reactive oxygen species and other indicators of oxidative stress [29, 32].

SOD is a potent protective enzyme that can selectively scavenge O_2_^∙^ into H_2_O_2_, which is further converted by CAT to harmless H_2_O and O_2_ molecules. Under ineffective antioxidant enzyme status, lipid peroxidation in the cellular and subcellular membranes is the inevitable outcome of ROS injury. A decrease in the activity of superoxide dismutase and catalase could result in the decreased removal of superoxide and hydrogen peroxide radicals, which brings about harmful events that can lead to the neuronal tissue damage.

PTZ-induced convulsions in this study significantly reduced the levels of catalase and superoxide dismutase while increasing lipid peroxidation products, which is indicative of significant oxidative stress in the brain tissue. MAE or diazepam pretreatment resulted in decrease in malondialdehyde (MDA) levels in brain tissue. The activities of superoxide dismutase (SOD) and catalase (CAT) were also increased in both treatment groups.

Pretreatment with MAE showed increased activity of these enzymes, which suggests that MAE may have ability to prevent the deleterious effects induced by free radicals. Lipid peroxidation is widely used to indicate oxidative injury in diseases [33]. A significant decrease in lipid peroxidation in the brain tissue of MAE- and diazepam-treated animals suggests some degree of protection from the direct oxidative insults.

Taken together, the data support a role for MAE in attenuation of neuronal damage after seizure attacks, in part at least, by inhibition of oxidative stress injuries. It is possible that the observed effect is a result of a complex interaction of different compounds in the extract. Interaction with the GABAergic and nitricoxidergic systems may not be an exhaustive list of the mechanism(s) for the anticonvulsant activity registered; hence, separation of active compounds and the specific activity and mechanisms thereof should be pursued in future research. However, the current evidence identified in our research gives some credence to the plants use in the management of epilepsy.

## 5. Conclusion

Findings of the present study suggest that the petroleum ether/ethyl acetate fraction of* Maerua angolensis* DC. stem bark has anticonvulsant activity at least in part through GABAergic and NO-cGMP pathway. Additionally, the extract exhibits comparable* in vitro* and* in vivo *antioxidant activities that might play a protective role in states of oxidative stress.

## Figures and Tables

**Figure 9:**
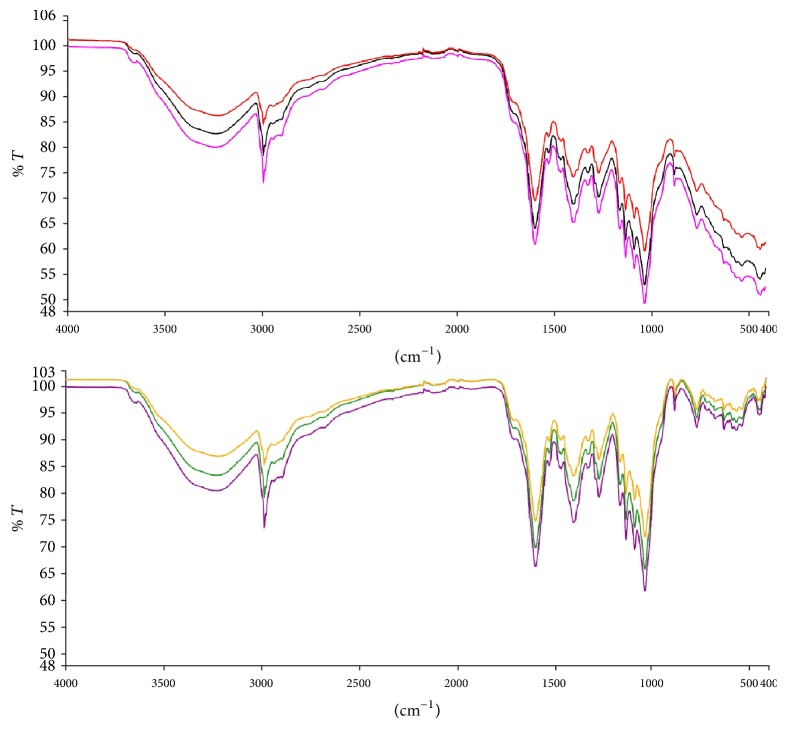
Original spectra of the petroleum ether/ethyl acetate fraction of* Maerua angolensis *DC. stem bark (baseline uncorrected versus baseline corrected).

**Table 1:** Peak tables for IR spectra of the petroleum ether/ethyl acetate fraction of* Maerua angolensis *stem bark.

Peak	*X* (cm^−1^)	*Y* (% *T*)
1	3226.52	82.82
2	2980.94	78.51
3	2100.15	98.53
4	1585.85	64.02
5	1515.39	78.99
6	1454.94	77.64
7	1386.52	68.83
8	1310.96	75.08
9	1258.88	70.16
10	1150.77	67.59
11	1120.87	61.74
12	1076.32	59.83
13	1023.17	52.81
14	869.99	74.68
15	755.57	66.71
16	523.8	56.6
17	430.93	53.96

1	3224.35	86.36
2	2980.96	84.61
3	2099.5	98.76
4	1585.79	69.48
5	1515.51	82.17
6	1454.78	81.33
7	1386.55	74.32
8	1310.69	78.91
9	1258.95	74.98
10	1150.65	73.01
11	1120.85	67.62
12	1076.39	66
13	1022.95	59.51
14	869.99	78.29
15	755.56	71.36
16	523.2	62.18
17	430.37	59.83

1	3227.3	80.11
2	2980.92	73.02
3	2103.82	97.56
4	1585.89	60.85
5	1515.37	76.86
6	1455	75.17
7	1386.39	65.15
8	1311.06	72.64
9	1258.82	67.03
10	1150.79	63.95
11	1120.87	58.23
12	1076.26	56.09
13	1023.43	49.11
14	869.93	72.48
15	755.56	64.08
16	523.45	53.54
17	427.16	50.8
18	407.14	51.61

## Abbreviations

5-HT: 5-Hydroxytryptamine
ANOVA: Analysis of variance
CAT: Catalase
cGMP: Cyclic guanosine monophosphate
CNS: Central nervous system
DPPH: 2,2-Diphenyl-1-picrylhydrazyl
DTNB: (5,5′-Dithiobis (2-nitrobenzoic acid))′
Dzp: Diazepam
EDTA: Ethylenediaminetetraacetic acid
EPM: Elevated-plus maze
FT-IR: Fourier transform-infrared spectroscopy
Fmz: Flumazenil
GABA: Gamma amino butyric acid
GABA_A_: GABA receptor type A
i.p.: Intraperitoneal
IR: Infrared spectroscopy
KNUST: Kwame Nkrumah University of Science and Technology
MDA: Malondialdehyde
MAE: * Maerua angolensis* extract
NADH: Nicotinamide adenine dinucleotide
NO: Nitric oxide
PTZ: Pentylenetetrazole
p.o.: Per os
PMS: Phenazine methosulfate
ROS: Reactive oxygen species
s.c.: Subcutaneous
SEM: Standard error of mean
SOD: Superoxide dismutase
TBA: Thiobarbituric acid
TBARS: Thiobarbituric acid-reactive species
TCA: Trichloroacetic acid.

## Additional Points

The chemical compounds studied in this article are as follows: diazepam (PubChem CID: 3016), pentylenetetrazole (PubChem CID: 5917), sildenafil citrate (PubChem CID: 62853), flumazenil (PubChem CID: 3373), adrenaline (PubChem CID: 122037), trichloroacetic acid (PubChem CID: 6421), thiobarbituric acid (PubChem CID: 2723628), sodium dodecyl sulphate (PubChem CID: 3423265), sodium chloride (PubChem CID: 5234), Tween 80 (PubChem CID: 443315), n-butanol (PubChem CID: 263), Triton-X 100 (PubChem CID: 5590), L-arginine (PubChem CID: 6322), DTNB (5,5′-Dithiobis (2-nitrobenzoic acid)) (PubChem CID: 6254), nitro blue tetrazolium (NBT) (PubChem CID: 9282), phenazine methosulfate (PMS) (PubChem CID: 9286), ethylenediaminetetraacetic acid (EDTA) (PubChem CID: 6049), DPPH (2,2-diphenyl-1-picrylhydrazyl) (PubChem CID: 2735032), NADH (nicotinamide adenine dinucleotide) (PubChem CID: 439153), and Tris-HCl (PubChem CID: 93573).
